# Unravelling the Mechanisms of Oxidised Low-Density Lipoprotein in Cardiovascular Health: Current Evidence from In Vitro and In Vivo Studies

**DOI:** 10.3390/ijms252413292

**Published:** 2024-12-11

**Authors:** Sahsikala Thangasparan, Yusof Kamisah, Azizah Ugusman, Nur Najmi Mohamad Anuar, Nurul ‘Izzah Ibrahim

**Affiliations:** 1 Department of Pharmacology, Faculty of Medicine, Universiti Kebangsaan Malaysia, Jalan Yaacob Latif, Bandar Tun Razak, Cheras 56000, Kuala Lumpur, Malaysia; p126086@siswa.ukm.edu.my (S.T.); kamisah_y@yahoo.com (Y.K.); 2 Cardiovascular and Pulmonary Research Group, Universiti Kebangsaan Malaysia, Bangi 43600, Selangor, Malaysia; dr.azizah@ppukm.ukm.edu.my (A.U.); nurnajmi@ukm.edu.my (N.N.M.A.); 3 Department of Physiology, Faculty of Medicine, Universiti Kebangsaan Malaysia, Jalan Yaacob Latif, Bandar Tun Razak, Cheras 56000, Kuala Lumpur, Malaysia; 4 Programme of Biomedical Science, Center for Toxicology & Health Risk Studies (CORE), Faculty of Health Sciences, Universiti Kebangsaan Malaysia, Jalan Raja Muda Abdul Aziz, Kuala Lumpur 50300, Malaysia

**Keywords:** oxidised LDL, cardiovascular health, atherosclerosis, inflammatory

## Abstract

Cardiovascular diseases (CVD) are the number one cause of death worldwide, with atherosclerosis, which is the formation of fatty plaques in the arteries, being the most common underlying cause. The activation of inflammatory events and endothelium dysfunction are crucial for the development and pathophysiology of atherosclerosis. Elevated circulating levels of low-density lipoprotein (LDL) have been associated with severity of atherosclerosis. LDL can undergo oxidative modifications, resulting in oxidised LDL (oxLDL). OxLDL has been found to have antigenic potential and contribute significantly to atherosclerosis-associated inflammation by activating innate and adaptive immunity. Various inflammatory stimuli such as interleukin-6 (IL-6), tumour necrosis factor-alpha (TNF-α) and intercellular adhesion molecule 1 (ICAM-1) play major roles in atherosclerosis. To date, studies have provided valuable insights into the role of oxLDL in the development of atherosclerosis. However, there remains a gap in understanding the specific pathways involved in this process. This review aims to provide and discuss the mechanisms by which oxLDL modulates signalling pathways that cause cardiovascular diseases by providing in vitro and in vivo experimental evidence. Its critical role in triggering and sustaining endothelial dysfunction highlights its potential as a therapeutic target. Advancing the understanding of its atherogenic role and associated signalling pathways could pave the way for novel targeted therapeutic strategies to combat atherosclerosis more effectively.

## 1. Introduction

Cardiovascular diseases (CVD) refer to any disorder that affects the heart and blood vessels of the vascular system, which is a major public health concern. They have accounted for more than 800,000 deaths every year from 2021 [1]. The vascular system consists of various cell types, including smooth muscle cells and endothelial cells (ECs). Positioned within the vessel lumen, ECs maintain direct contact with circulating blood cells, playing a pivotal role in regulating blood pressure and orchestrating leukocyte trafficking [2]. However, in numerous pathological conditions of CVD, the normal physiological function of blood vessels becomes compromised due to inflammatory responses, oxidative stress and aberrant expression of surface molecules on ECs. These disruptions contribute to the progression of vascular dysfunction, emphasising the intricate interplay between cellular components and environmental factors in the context of vascular health [3].

Coronary artery disease is the most common type of heart disease, characterised by the narrowing or blockage of the coronary arteries due to the build-up of plaque, a condition known as atherosclerosis. Atherosclerosis can be defined as a chronic inflammatory response in the walls of arteries, in which the inner lining of the artery becomes damaged and then accumulates fatty deposits and inflammatory cells, resulting in the narrowing of the artery. The narrowing of the arteries reduces the amount of blood and oxygen that can reach the heart, resulting in chest pain (angina) and potentially leading to a myocardial infarction [4]. From this, one can understand that atherosclerosis is a prevalent risk factor for CVD, serving as the root cause of numerous heart-related diseases, including coronary artery disease, stroke, and peripheral artery disease [1].

The immune system plays an important role in the development, progression, and complications associated with atherosclerosis. Innate and adaptive immune responses are linked with the progression of the disease [5]. The atherogenic process begins with the accumulation of various plasma lipoproteins, particularly in the subendothelial space, where flow disturbances and endothelial dysfunction occur. Low-density lipoprotein (LDL) is particularly well-documented in this process, with its accumulation linked to traditional risk factors such as smoking, hypertension and metabolic disorders. Within the intima, LDL undergoes oxidative modifications by reactive oxygen species (ROS), which facilitate the uptake of oxidised LDL (oxLDL) into macrophages [6]. The involvement of macrophages highlights a crucial aspect of innate immunity, with dendritic cells (DCs) also playing an essential role as part of the immune response. OxLDL can promote DCs’ maturation and induce the expression of major histocompatibility II (MHC-II), thus bridging innate and adaptive immunity. Through this process, DCs can process oxLDL and present it as an antigen to T-cells, which further activates the immune response [7]. T-cells are a part of adaptive immunity; therefore, adaptive immunity takes place at this stage. OxLDL is an antigen for T-cell activation, particularly through DC-mediated presentation. CD4+ and CD8+ T-cells can both recognise oxLDL, with CD4+ T-cells responding to specific peptide epitopes on oxLDL [8]. These T-cells drive atherogenic inflammation and can increase lesion development. In the meantime, B-cells, another member of the adaptive immunity, exert a humoral immune effect by producing antibodies against oxLDL, with IgM antibodies showing a protective role by neutralising oxLDL, whereas IgG antibodies can exacerbate inflammation [9]. Therefore, oxLDL plays a role as a potent antigen in atherosclerosis by employing and activating innate, adaptive and humoral immune responses.

In a review, Poznyak et al. (2021) concluded that oxLDL plays a significant role in the progression of atherosclerosis owing to its effects on lipid accumulation and inflammation within the vascular system [10], which eventually lead to endothelial cell (EC) dysfunction. EC dysfunction is a pivotal factor during the initiation and progression of CVD, contributing to atherogenesis and the formation of arterial plaques [11]. Various stimuli, including inflammatory factors such as interleukin-6 (IL-6), tumour necrosis factor-alpha (TNF-α) and intercellular adhesion molecule 1 (ICAM-1) induce dysfunction in ECs and foam cell formation, causing inflammation in the blood vessel walls and driving the development and advancement of CVD [12]. Inflammation in the blood vessel walls is closely related to the oxidation of LDL, which subsequently results in atherogenic properties. The LDL particles undergo several modifications that alter the size, density and chemical properties within the blood flow and vascular wall, ultimately being oxidised by ROS [13].

Indeed, oxidative stress is strongly associated with inflammatory responses and is reciprocally related [14]. OxLDL is more likely to adhere to the inner lining of artery walls, contributing to plaque formation. OxLDL, during plaque formation, could activate endothelial cells to induce the expression of several cell surface adhesion molecules that in turn mediate the rolling and adhesion of blood leukocytes (monocytes and T-cells). Following adhesion to the endothelium, leukocytes migrate into the intima in response to chemokines [15] that play important roles in atherosclerosis development. Therefore, the accumulation of oxLDL in the artery walls triggers an inflammatory response, which results in the recruitment of immune cells and the formation of fatty deposits, smooth muscle cells and other components of the plaque. Over time, this can cause hardening and narrowing of the arteries, reducing blood flow and increasing the risk of complications, such as heart attack and stroke. These fundamental processes proved that the oxidation of LDL is a critical step in the development of atherosclerosis [16,17]. Indeed, the levels of oxLDL are positively associated with the severity of coronary atherosclerosis in metabolically dysfunctional pathologies frequently associated with CVD, including diabetes mellitus [18]. Furthermore, elevated oxLDL levels have been linked to a higher risk for recurrent myocardial infarction after adjustment of age, gender, diabetes mellitus, previous myocardial infarction and previous angina [19]. It has also been found that elevated oxLDL levels are significantly associated with a higher presence of CVD in patients with chronic inflammatory conditions compared to chronically inflamed individuals without CVD [20]. In a recent study by Saarinen et al. (2024), it was demonstrated that men with metabolic syndrome who had high-sensitive C-reactive protein (hs-CRP) of more than 1.0 mg/L had a higher risk for CVD and all-cause mortality than those with hs-CRP of less than 1.0 mg/L [21]. These studies demonstrated that inflammation is one of the factors that can contribute to CVD.

To date, studies have given insights into the role of oxLDL in atherosclerosis; however, the specific relationships within its signalling pathways in particular remain unclear. This review article aims to present current evidence from in vitro and in vivo studies on atherosclerotic events, which could offer foundational insights into the signalling pathways of oxLDL in cardiovascular health, with a particular focus on endothelial dysfunction and atherosclerosis.

## 2. Oxidised Low-Density Lipoproteins, Oxidative Stress and Inflammation in Foam Cell Formation

Lipoproteins are large macromolecular complexes comprising lipids and proteins that are responsible for transporting lipids through the bloodstream and delivering them to tissues. Among these, LDLs act as the primary carriers of cholesterol, containing mainly cholesterol esters and free cholesterol [22]. The composition and distribution of LDL subfractions vary among individuals depending on genetic and environmental factors [23]. Notably, small and dense LDL particles are associated with an increased risk of cardiovascular disease, and patients with cardiovascular disease tend to exhibit higher levels of these particles compared to control subjects [24]. Under normal physiological conditions, the body maintains oxidative balance. However, increased reactive oxygen species (ROS) production can lead to the oxidation of various LDL components such as phospholipids, cholesterol esters and polyunsaturated fatty acids that lead to the formation of oxLDL [25].

Oxidative stress, defined as an imbalance between ROS production and accumulation, contributes to intracellular ROS accumulation and other free radicals implicated in the onset and progression of diseases such as diabetes, obesity, cancer, neurodegenerative diseases and CVD [26]. In atherosclerosis, the oxidation of LDL particles within the vascular endothelium is considered an initial step in the formation of atherosclerotic plaques [10,27]. Oxidative modification transforms LDL into atherogenic particles that trigger inflammatory responses. When oxLDL is taken up by macrophages, it initiates a cascade of bioactivity that contributes to atherosclerotic lesion development.

The relationship between total serum cholesterol and coronary heart disease mainly reflects the correlation between LDL cholesterol levels and total cholesterol in most people [28]. Elevated circulating LDL levels are often found in diets high in saturated fat or cholesterol [29,30]. LDL, which contains primarily apoB-100, is the major carrier of cholesterol and carries it to various tissues, including the adrenal glands, gonads, muscle and adipose tissue [31]. These tissues possess LDL receptors on their plasma membranes that recognise apoB-100. The LDL particles are taken up by receptor-mediated endocytosis, where the digestion of lipoprotein in lysosomes will occur. Subsequently, the cholesterol is released into the cell to be used for membrane synthesis, or it is stored as a cholesterol ester [32]. Circulating LDLs are also taken up by the endothelial cells of the artery wall, traverse through it and become trapped in the arterial intima. In the intima, they may undergo oxidation or other biochemical modifications, be ingested by macrophages and promote atherogenesis [28]. This process induces phenotypic changes in endothelial cell surfaces, driven by the production of intracellular ROS [10,27].

Macrophages that accumulate triglycerides or cholesterol esters in these lesions are referred to as “foam cells” owing to the appearance of large intracellular lipid droplets resembling spongy foam. Although foam cells primarily originate from macrophages, other cell types, such as endothelial and smooth muscle cells, also accumulate lipids to a lesser extent. LDL, the main cholesterol carrier in the bloodstream, has its concentration regulated by very low-density lipoprotein (VLDL) secretion from the liver and LDL receptor-mediated clearance from circulation. However, when LDL undergoes oxidative modification, the oxidised form (oxLDL) loses its ability to bind to LDL receptors and instead attaches to scavenger receptors. The expression of LDL receptors is controlled by the cholesterol content within cells, preventing excessive cholesterol accumulation. In contrast, scavenger receptors on macrophages and other cells continue to take up oxLDL, even when cholesterol accumulates, resulting in the formation of foam cells [33].

**Figure 1:**
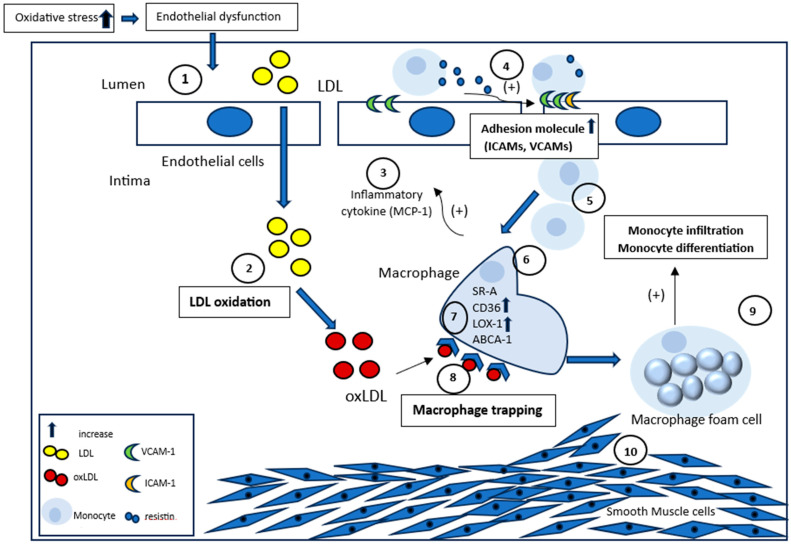
Macrophage foam cell formation and inflammation in cardiovascular health. (1) Elevated ROS production and oxidative stress induce endothelial dysfunction and alter endothelium permeability, causing LDL entry into the arterial intima layer. (2) LDL in the intima layer undergoes modification and oxidation to form oxLDL, which (3) activates endothelial cells to produce chemoattractant factors and cytokines, such as MCP-1, which promotes the recruitment of monocytes from the lumen into the arterial intima. (4) Resistin, which is secreted by monocytes, increases the adhesion markers ICAM-1 and VCAM-1 in endothelial cells. (5) Thus, monocyte binds together and infiltrates the arterial intima. M-CSF (cytokine) can differentiate infiltrated monocytes into macrophages. (6) Monocytes are differentiated into macrophages which subsequently incorporate the modified LDL, generating a foamy appearance within the macrophages, which is recognised as foam cells. (7) The differentiated macrophages express a scavenger receptor such as SR-A, CD36 and LOX-1. (8) OxLDL in the intima can attach to differentiated macrophages, leading to a process known as macrophage trapping. Ultimately, cholesterol transporter (ABCA-1) transforms these macrophages into foam cells. (9) The resulting macrophage foam cells continue to release pro-inflammatory cytokines to promote the monocyte infiltration process, which in turn increases inflammation through apoptosis and necrosis. (10) The inflammatory cytokines promote the infiltration and proliferation of smooth muscle cells from the media into the arterial intima, leading to the thickening of the arterial walls and the transformation of the fatty streak into a stable plaque. ABCA-1: ATP-binding cassette transporter; CD36: cluster of differentiation 36; LDL: low-density lipoproteins; LOX-1: lectin-like oxidised low-density lipoprotein receptor; MCP-1: Monocyte chemoattractant protein-1; ROS: reactive oxygen species; SR-A: scavenger receptor-A. This figure is modified from Chae et al. (2019) [36] and Ooi et al. (2017) [37].

The formation of foam cells derived from macrophages in the intima region is a crucial step for the occurrence and development of atherosclerosis. The foam cells are identified as the major hallmark of early-stage atherosclerotic lesions. OxLDL could initiate the inflammation process in blood vessels through the activation of macrophages and other cells (Figure 1) [34]. OxLDL has been shown to trigger a persistent proatherogenic phenotype in macrophages through epigenetic histone modifications that lead to increased pro-inflammatory cytokine production and the formation of foam cells [35]. Primarily, oxLDL accumulates within the blood vessel intima, causing endothelial dysfunction and subsequent release of chemokines, including MCP-1 (Monocyte Chemoattractant Protein-1). This attracts circulating monocytes to the inflamed site, where they attach to endothelial cells through a process called “rolling”. Following attachment, monocytes release molecules like resistin, which upregulate adhesion molecules like VCAM-1 and ICAM-1 on endothelial cells, facilitating monocyte infiltration into the intima. Once inside, cytokines like M-CSF drive the differentiation of monocytes into macrophages. These macrophages express scavenger receptors such as SR-A, CD36 and LOX-1, allowing them to bind oxLDL and become trapped within the intima. Subsequently, the trapped macrophages internalise oxLDL and later transform into foam cells laden with lipid droplets, characteristic of early atherosclerotic lesions.

Cholesterol transporters like ABCA-1 and ABCG-1 regulate cholesterol efflux from foam cells, while dysregulation can lead to further foam cell formation. Meanwhile, foam cells perpetuate inflammation by secreting pro-inflammatory cytokines, thereby promoting the recruitment of more monocytes and exacerbating the inflammatory response, ultimately contributing to the progression of atherosclerosis through processes like apoptosis and necrosis. Overall, the process of macrophage foam cell formation is a critical step in the pathogenesis of atherosclerosis, linking lipid accumulation, inflammation and immune cell recruitment in the development of arterial plaques [36]. Macrophages sustain local inflammation by releasing proinflammatory molecules and interacting with vascular smooth muscle cells, which amplifies the response and promotes lipoprotein retention, perpetuating the inflammatory cycle [10].

## 3. Experimental Evidence of Oxidised Low-Density Lipoprotein Effects in Cardiovascular Health

### 3.1. Direct Effects on Various Types of Cells

The direct effects of oxLDL have been previously investigated in different cells, including mononuclear cells, endothelial cells, macrophages and smooth muscle cells (Table 1). In human peripheral blood mononuclear cells (hPBMC), it has been shown that oxLDL can reduce cell viability but increase free radical formation and inflammation in the mononuclear cells [38]. For endothelial cells, studies have used human umbilical vein endothelial cells (HUVECs), mouse lung endothelial cells and human aortic endothelial cells. Similarly, oxLDL also reduced cell viability [39], increased cell proliferation [40] and increased cell migration in endothelial cells [41]. In the early stages of atherosclerosis, disrupted blood flow contributes to the dysregulation of endothelial cell proliferation and turnover. Due to this, atherosclerosis is characterised by cellular processes that promote EC turnover and induce proliferation of the activated endothelium [42,43]. In addition, oxLDL can increase reactive oxygen species (ROS) in endothelial cells [39,44,45] and reduce superoxide dismutase (SOD) activity [39]. In addition to this, oxLDL also increases endothelial adhesion molecules, such as intercellular adhesion molecules (ICAM-1) and vascular adhesion molecules (VCAM-1) [46,47,48]. Increased apoptosis of endothelial cells has also been observed in several studies [39,41].

Macrophages have been introduced to determine the effect of oxLDL related to cardiovascular health. In a study by Guo et al. (2018), oxLDL was reported to reduce cell viability, which is parallel to increased cell apoptosis [49]. Similar to mononuclear cells and endothelial cells, oxLDL was reported to have a direct effect on inflammation and oxidative stress, as evidenced by an increase in IL-1β levels and ROS [50]. In addition, oxLDL demonstrated a direct effect on oxidative stress via increased intracellular MDA and ROS production as well as a reduction in SOD activity [51]. In addition to this, oxLDL has been tested in smooth muscle cells, in which it was reported to increase bone sialoprotein expression [52].

### 3.2. Direct Effects on Animals

The effects of oxLDL on cardiovascular health have been studied in various animal strains, including mice (C57 BL/6, ApoE KO, LOX-1 transgenic, CD 36 KO) and Wistar rats (Table 2). The effects of oxLDL have been shown by the modulation of the immune system as evidenced by increased T-cell tolerance [53] and IFNγ production. In addition to ROS, oxLDL has also affected immune response in in vivo studies. In a study by Nicoletti et al. (2000), oxidised LDL that was intraperitoneally injected into mice pups within the first 24 h of life demonstrated neonatal tolerance to oxidised LDL. The mice pups were given birth to by pregnant ApoE KO female mice and male mice injected with oxLDL. In addition, the mice pups fed with a Western diet at 5 weeks of age also demonstrated high susceptibility to atherosclerosis and high T-cell tolerance compared to the control group [53]. This study supports the accumulating evidence suggesting that inflammatory responses and immunoregulation play a crucial role in atherosclerosis development [54].

In a study by Steinmetz (2015) using male C57BL/6 apoE knockout mice injected with oxLDL, increased IFN-γ production was demonstrated compared to the control group injected with PBS. OxLDL-injected mice also showed increased lesion size of atherosclerotic plaques in the aortic roots [55]. IFN-γ is important for innate and adaptive immunity, which exerts its effects via activation of macrophages, natural killer cells and B-cells. Macrophage activation by IFN-γ leads to the production of other pro-inflammatory cytokines like tumour necrosis factor (TNF)-α and IL-6, oxygen radicals and metalloproteinases that subsequently promote atherosclerosis [56].

In addition, oxLDL also has a direct effect on the kidney, which has been associated with the production of nephrotoxic properties [57]. As well as affecting the immune system, oxLDL has also been associated with abnormal renal excretion. In a recent study by Dabkowski et al. (2024), injection of OxLDL into Wistar rats affected renal function by increasing urinary albumin excretion by approximately 28% but did not affect nephrin excretion. The study indicates that oxLDL interferes with the renal handling of albumin and leads to the development of albuminuria, therefore increasing inflammatory events and atherogenic events [57]. In addition, the study also demonstrated that oxLDL-injected rats had higher chances of nephrotoxic properties that predict for cardiovascular events [57].

Similar to the effect on cells, oxLDL effects in animals also include increasing reactive oxygen species [58,59] and inflammatory marker CD36 [60]. For instance, a study by Zhang et al. (2016) reported that injection of oxLDL for three days increased bone marrow and blood intracellular and extracellular ROS production in wild-type C57 BL/6 mice [59]. In a study by Cui et al. (2015), a single dose of non-atherogenic human native LDL was intravenously injected into male C57BL/6 mice. Findings indicated that serum oxLDL was detected within 30 min following the human native LDL injection and reached the peak level in three hours compared to the control (human Sat-LDL). A significant increase in ROS production was detected in the human native LDL group [58]. The study suggests that following injection of human native LDL into mice, human oxLDL was rapidly detected in blood. Several previous studies have established that an increase in ROS accumulation contributes to an imbalance in the ambient redox state, promoting endothelial cell injury and facilitating oxidation of native LDL [22,61]. LDL oxidative modification predominantly occurred in macrophages and/or smooth muscle cells during the process of foam cell formation [62,63].

The effect of oxLDL has also been studied on the cluster of differentiation-36 (CD36), an atherogenic protein which is recognised for its in vitro ability to take up oxLDL in macrophages. In a study by Luangrath et al. (2008), the physiological role of CD36 in native LDL and oxLDL metabolisms in mice was investigated, and it was shown that CD36 favoured oxLDL clearance, observed when the clearance of mildly oxidised LDL and fully oxidised LDL was delayed. This indicates that CD36 enhances the holoparticle uptake (complete particle internalisation) of oxLDL by the liver, helping to remove oxLDL from the bloodstream. Moreover, it was also demonstrated that CD36 hinders the clearance of native LDL. While it enhances the removal of oxLDL, it slows down the clearance of native LDL, making it a double-edged sword in atherogenesis. This effect may contribute to the atherogenic properties of CD36 by increasing the circulation time of native LDL, which can be oxidised and contribute to plaque formation. Therefore, the study concluded that oxLDL metabolism and clearance are accelerated by CD36, which might be protective, but it simultaneously slows down native LDL clearance, potentially increasing atherosclerosis risk [60].

**Table 2:** In vivo experimental evidence for oxidised low-density lipoprotein in atherosclerosis.

Animal Strain	Experimental Groups and Concentration	Findings	Study
Male mice C57 BL/6	Animals divided into two groups: Group I: Control (human Sat-LDL) Group II: human native LDL (50 μg) was injected to mice	Following human native LDL injection: ↑ Serum ox-LDL level by 30 min (rapid conversion to oxLDL) Reached peak level in 3 h Undetectable in 12 h Detectable level of both intracellular and extracellular ROS Following human Sat-LDL injection: Absent in vivo ROS production	Cui et al. (2015) [58]
Wild type C57 BL/6 mice	Animals divided into two groups: Group I: Control (injected with 50 μg of PBS for 3 days) Group II: oxLDL (mice were injected with 50 μg of oxLDL for 3 days)	oxLDL injected mice: ↑ bone marrow and blood extracellular and intracellular ROS	Zhang et al. (2016) [59]
Offspring of Pregnant ApoE KO female mice and male mice	Animals divided into two groups: Group I: Control In first 24 h of life: Injected with PBS Group II: OxLDL In first 24 h of life: Injected with 60–80 μg oxLDL Both groups were fed with a Western diet at 5 weeks of age	Injection of oxidised LDL: ↓ immune response to oxLDL ↑ susceptibility to atherosclerosis ↑ T-cell tolerance	Nicoletti et al. (2000) [53]
Male C57BL/6 apoE knockout mice	Group I: Control (injected with PBS) Group II: oxLDL was injected to apoE KO mice	Injection of oxLDL: ↑ IFNγ production ↑ atherosclerotic plaques lesion size in the aortic roots	Steinmetz et al. (2015) [55]
Male Wistar rats	Animals divided into two groups: Group I: Control (injected with PBS) Group II: oxLDL (injected with 4 mg of oxLDL protein/kg of b.w.)	Injection of oxLDL: ↑ Urine albumin ↑ inflammatory events ↑ atherogenic events ↑ chances nephrotoxic properties	Dąbkowski et al. (2024) [57]
C57BL/6 male mice	Animals divided into two groups: Group I: control (injected with 20 ug of Dil-nLDL Group II: oxLDL (injected with 20 μg of Dil-oxLDL	oxLDL group: ↓ in cell surface LRP6 ↑ in cell surface LRP5	Wang et al. (2018) [64]
Endothelial-specific LOX-1 transgenic and wild type male mice	Animals divided into two groups: Group I: WT mice (injected with 63 mg/kg of PBS) Group II: specific LOX-1 TG oxLDL (injected with 40 mg/mL of oxLDL)	oxLDL LOX-1 transgenic mice: ↑ LOX-1 overexpression in isolated carotid arteries ↓ TF expression ↑ Oct-1 in mRNA and protein level in carotid arteries ↑ Extracellular protein kinase ERK1/2 phosphorylation Mice fed with HCD for 6 weeks starting at 6 weeks of age: ↑thrombus formation ↑ oxLDL levels in plasma	Akhmedov et al. (2017) [65]
CD36 wild-type mice	Group I: wild type (no alteration) Group II: CD36 KO Group III: SR-BI KO Group IV: CD36/SR-BI dKO * All mice were injected with either native or mildly oxLDL or oxLDL	CD36/SR-BI dKO injected with OxLDL: ↑ cholesterol, triglycerides and FFA ↑ CD36 CD36 mediates oxLDL holoparticle uptake	Luangrath et al. (2008) [60]

Abbreviations: b.w.: body weight; CD36: cluster of differentiation-36; ERK1/2: extracellular signal-regulated kinases; IFNγ: interferon gamma; KO: knockout; LRP 5/6: low-density lipoprotein receptor-related protein 5/6; LOX-1: lectin-type oxidised LDL receptor 1; Lox-1 TG: lox-1 transgenic; PBS: phosphate buffered saline; ROS: reactive oxygen species; Sat-LDL: saturated LDL; SR-BI: scavenger receptor class B, type I; TF: tissue factor; Oct-1: octamer transcription factor-1. ↑: increase ; ↓: decrease; *: contains non radiolabelled lipoproteins and lipoproteins radiolabelled with ^125^I or [^3^H]Cet.

## 4. Activation Pathways

**Figure 2:**
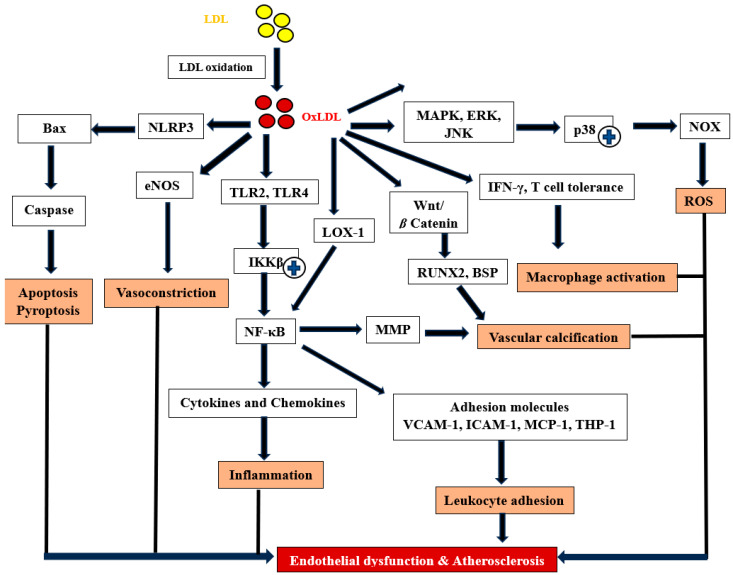
OxLDL affects various signalling pathways which are associated with the pathogenesis of atherosclerosis, including apoptosis, pyroptosis, inflammation, vascular calcification, leukocyte adhesion and reactive oxygen species. Bax: Bcl-2 Associated X-protein; BSP: bone sialoprotein; eNOS: endothelial nitric oxide synthase; IFN-γ: interferon gamma; IKKβ: IκB kinase β; LOX-1: lectin-type oxidised LDL receptor-1; MAPK: mitogen-activated protein kinase; MCP: monocyte chemoattractant protein-1; MMP: matrix metalloproteinase; NFκB: Nuclear factor kappa-light-chain-enhancer of activated B; NLRP3: NLR family pyrin domain containing 3; Runx-2: Runt-related transcription factor-2; TLR: Toll-like receptor; Wnt/β Catenin: Wingless/Beta Catenin; 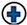: activated.

Based on the in vitro and in vivo studies discussed earlier in Section 3 and Section 4, several signalling pathways have been identified. Table 3 summarises the functions and key roles of each signalling pathway, while Figure 2 illustrates their interconnected relationships within the pathogenesis of endothelial dysfunction and atherosclerosis.

### 4.1. Nuclear Factor Kappa-Light-Chain-Enhancer of Activated B-Cells (NF-κB)

NF-κB is composed of a family of transcription factors responsible for inflammation, immunity, cell proliferation, differentiation and survival [73]. The activation of NF-κB requires the phosphorylation and degradation of the inhibitor of NF-κB proteins (IκBs), which is triggered by two kinases: IKK α and IKK β [73]. In a study by Yurdagul et al. (2016), oxLDL showed high IKKβ activation (two-fold increase in IKKβ activation at 30 min). In addition, endothelial cells transfected with IKKβ siRNA and that had been oxLDL-induced demonstrated a reduction in NF-κB phosphorylation, nuclear translocation and VCAM-1 expression. This observation indicates that classical NF-κB signalling through IKKβ mediates the pro-inflammatory responses to oxLDL [44].

NF-κB can regulate the transcription of many genes including cytokines, chemokines and adhesion molecules [74]. This fact is in accordance with several previous studies demonstrating increased gene and protein expression of the cytokines, chemokines and adhesion molecules. For instance, oxLDL has been shown to increase the adhesion of THP-1 to monocytes rapidly compared to untreated control cells in the adhesion assay. Following this, HUVECS induced with oxLDL increased the protein expression of adhesion-related genes, including VCAM-1, ICAM-1, MCP-1 and IL-8 [48]. Meanwhile, in a study by Saji et al. (2018), the exposure of endothelial cells to oxLDL also led to significantly increased expressions of adhesion assay and inflammatory markers including iNOS, TNF-α, IL-6 and VCAM-1 compared to the control group [38]. A direct relationship between inflammation and adhesion of monocytes during atherosclerosis development has been observed in previous studies, in which miRNAs that linked to inflammation such as miRNA-155 and miRNA-103 demonstrated increased adhesion of monocytes to endothelial cells [75,76]. A direct relationship between inflammation and monocyte adhesion had also been highlighted in a previous study by Zhang et al. (2021) in which supplementation of oxLDL to HUVECs showed increased mRNA and protein expression levels of TNF-α, IL-1β and IL-6 compared to the control group. Meanwhile, analysis of RT-PCR displayed increased ICAM-1 and VCAM-1 in oxLDL-treated HUVECs [46].

### 4.2. Toll-like Receptors (TLRs)

Innate immunity serves as the first line of defence against invading pathogens. A family of TLRs acts as primary sensors in detecting a wide range of microbial components and initiating innate immune responses. The TLR signalling pathways could mediate the activation of the NF-κB [77]. In a study by Zhang et al. (2021), oxLDL was reported to modulate Toll-like receptors (TLR) that ultimately activated the transcription factor NF-κB. The upregulation of NF-κB has been linked to TLR4, as demonstrated by increased TLR4 mRNA expression in HUVECs treated with oxLDL. A hyper-inflammatory state was also recorded by the significant upregulation of mRNA and protein expression levels of TNF-α, IL-1β and IL-6, which was largely dependent on the TLR4/NF-κB signalling pathway [46]. The study suggests that the increased level of inflammatory markers following the oxLDL supplementation to the cells was associated with the modulation of TLRs. This study is supported by Bashkar et al. (2016), where the treatment of oxLDL on HUVECs significantly increased TLR-2 and TLR-4 mRNA and protein expression [47]. In addition, the Bashkar et al. study had shown that oxLDL activated nuclear translocation of the NF-κB p65 subunit and increased inflammatory cytokines, demonstrating that oxLDL could modulate TLR and NF-κB signalling pathways and regulate the expression of numerous inflammatory cytokine genes.

### 4.3. Mitogen-Activated Protein Kinase (MAPK)

OxLDL modulates mitogen-activated protein kinase (MAPK) cascades involved in the extracellular signal-regulated kinase (ERK), as evidenced by several studies [44,45,48,51]. ERK 1 and ERK 2 are evolutionarily conserved, ubiquitous serine-threonine kinases that regulate cellular signalling under normal and pathological conditions [78]. In a study by Yang et al. (2015), oxLDL-treated cells demonstrated increased phosphorylation levels of p38, JNK and ERK1/2 compared to untreated cells [51]. JNK and p38 activation are sensitive to ROS; hence, indicating ROS produced by NADPH oxidase is necessary for oxLDL-induced atherosclerosis conditions [79]. In addition, the increased ERK1/2 indicates that oxLDL modulates via ERK1/2, a member of the MAPK signalling pathway [65,80]. The MAPK signalling pathway also includes p38 and c-Jun-N-terminal kinase 1/2/3 (JNK1/2/3) among its members [81], which are also upregulated in the study.

Furthermore, the MAPK signalling pathway also involves ERK5, whose dysfunction is associated with cardiovascular diseases including atherosclerosis [82]. Deng et al. (2018) revealed that the supplementation of primary human umbilical vein endothelial cells for 24 h reduced phosphorylated ERK5, as observed via Western blot analysis [48]. Supporting this, a previous study by Le et al. (2013) reported that ERK5 knockout mice exhibited impaired vessel reactivity that could lead to endothelial dysfunction and atherosclerosis [83]. In addition, the supplementation of primary HUVECs also reduced the expression of endothelial nitric oxide synthase (eNOS) [48], which is a fundamental determinant of cardiovascular homeostasis, thus regulating systemic blood pressure, vascular remodelling and angiogenesis [84]. The MAPK signalling pathway is stimulated by oxLDL through the PI3K/AKT pathway, which plays a significant role in promoting apoptotic cell death [85]. An active form of AKT regulates cell proliferation and apoptotic responses [86]. This fact is supported by several studies demonstrating increased AKT phosphorylation in oxLDL-treated cells [39,40,46].

### 4.4. Nuclear Factor Erythroid 2-Related Factor 2 (NRF2)

Nuclear factor erythroid 2-related factor 2 (NRF2) is a transcription factor that regulates the cellular defence against toxic and oxidative insults [87]. Nrf2 has antioxidant effects that are closely linked to several cardiovascular diseases, primarily through its role in enhancing endothelial function [88]. This transcription factor is involved in regulating the biosynthesis, utilisation and regeneration of glutathione, thioredoxin and NADPH. In addition, Nrf2 is engaged in the production of reactive oxygen species via the mitochondria pathways and NADPH oxidase, helping to maintain cellular redox homeostasis [89]. In particular, oxLDL has been associated with a reduction in Nrf2 expression and a corresponding increase in ROS when introduced to HUVECs [39].

OxLDL could also regulate nicotinamide adenine dinucleotide phosphate (NADPH) oxidases (NOX). In a study by Yang et al. (2015) on mouse RAW264.7 macrophages, oxLDL-treated cells illustrated high expression of NOX2, Rac1, p47phox and p22phox compared to untreated cells [51]. NADPH acts as a substrate to convert molecular oxygen to ROS, while NADPH oxidases are enzymes that are comprised of membrane-associated cytochrome components (catalytic gp91phox (or Nox2) and regulatory p22phox subunits) and cytosolic components (p47phox, p67phox, p40phox and small GTPases). As a general source of ROS production in vascular cells, NOX enzymes play an important role in atherosclerosis and may serve as therapeutic targets for the treatment of cardiovascular diseases [90]. Therefore, this study highlights the potential of targeting NOX enzymes in therapeutic treatments, especially for atherosclerosis generally induced by oxLDL.

### 4.5. Nucleotide-Binding Domain, Leucine-Rich-Containing Family, Pyrin Domain-Containing-3 (NLRP-3) Inflammasomes

Inflammasomes are receptors or sensors of the innate immune system that trigger and promote inflammation. NLRP3, which is the best-characterised inflammasome [91], is upregulated in cardiovascular disorders including atherosclerosis, myocardial infarction, ischemic heart disease, chronic heart failure and hypertension [92]. OxLDL has been demonstrated to induce an inflammatory form of cell death, namely pyroptosis, which is triggered by certain inflammasomes. The inflammatory trigger by inflammasomes could lead to the activation of inactive cytokines like IL-18 and IL-1β [93]. In a previous study by Singh et al. (2019) using RAW264.7 mouse macrophages, oxLDL had increased the level of NLRP3 inflammasome, active caspase-1 and IL-1β. It also modulated the expression of BRCC36 deubiquitinating enzymes and released LDH [50], which is associated with pyroptotic cell death [94].

In addition to pyroptosis, NLRP3 inflammasome activation also contributes to apoptosis, necroptosis and ferroptosis. In a study by Liang et al. (2021), oxLDL-induced apoptosis in HUVECs resulted in increased protein expression of Bax and β-catenin compared to untreated cells [41]. A study by Lin et al. (2017) demonstrated that β-catenin overexpression induces apoptosis by downregulating survival pathways in cardiomyocytes. This effect was shown through pCMV-β-catenin plasmid transfection, which led to increased expression of apoptotic protein markers, including Bax, cytochrome c, caspase 3 and caspase 9, in neonatal rat cardiomyocytes [95]. The study could support the hypothesis that oxLDL can induce apoptosis via the NLRP3-Bax/β-catenin-caspase signalling pathway.

The endoplasmic reticulum (ER), a crucial organelle responsible for regulating fundamental cellular processes, contains stress signalling pathways that have been shown to affect NLRP3 inflammasome activation and contribute to atherosclerosis [96]. In a study by Guo et al. (2018), oxLDL supplementation was shown to increase protein expression of p-eIF2α (the active form of eIF2α), p-PERK (the active form of PERK) and p-IRE1α (the active form of IRE1α), as well as mRNA expressions of ATF4 and HERPUD1 [49]. Furthermore, in a study by Onat et al., 2019, it was shown that eIF2α phosphorylation was persistently observed in mouse and human atheroma, indicating the involvement of ER signalling in atherosclerosis development. In addition, the activated eIF2α induced a mitochondrial protease, Lon protease 1 (LONP1), resulting in greater mitochondrial oxidative stress and inflammasome activation in macrophages [97]. The study indicates that ER signalling could induce inflammasome activation regulated by the NLRP3 inflammasome.

### 4.6. Wingless/Beta Catenin (Wnt/β Catenin)

Activation of the Wnt/β-catenin pathway involves the binding of a Wnt ligand from the extracellular environment to two transmembrane proteins: a Frizzled protein acting as the receptor and a low-density lipoprotein receptor-related protein 5 or 6 (LRP 5/6) acting as a co-receptor [98]. This pathway plays a role in multiple aspects of vascular lesion development and progression including endothelial dysfunction, macrophage activation, proliferation and vascular smooth muscle cell migration [99]. WNT/β-catenin signalling has been established as essential for VSMC differentiation [100] in promoting vascular smooth muscle cells to osteogenic transdifferentiation and calcification through directly modulating Runx2 gene expression [101].

Vascular calcification is an essential stage in atherosclerosis, whereby vascular smooth muscle cells (VSMCs) synthesise many osteogenic factors such as bone sialoprotein (BSP) and runt-related transcription factor 2 (Runx2) [102]. The upregulation of osteogenic transcription factors typically involved in bone formation has emerged as a key regulator of adverse cellular events in the cardiovascular system. This phenomenon plays a significant role in driving cardiovascular pathology, particularly in conditions like vascular calcification and atherosclerosis [103]. Under pathological conditions, vascular smooth muscle cells (VSMCs) and other cell types within the cardiovascular system undergo phenotypic changes, transitioning from a contractile to an osteogenic-like state. According to a study by Farrokhi et al. (2015), an in vitro model of atherosclerosis generated by treating human VSMCs with oxLDL increased the expression of BSP and Runx2 genes and proteins nearly five-fold [52]. The study highlights that oxLDL could modulate the osteogenic transcription factors in the vascular smooth muscles during pathologic conditions, particularly in vascular calcification.

Meanwhile, a study by Wang et al. (2018) demonstrated that oxLDL modulated co-receptors of the Wnt/β-catenin pathway by increasing LRP 5 and decreasing LRP 6 in high cholesterol diet-fed mice injected with oxLDL [64]. In the development of atherosclerosis, LRP 5 levels were higher in advanced plaques compared to early lesions. In addition, LRP5 mRNA and protein levels were also increased when human monocytes and macrophages were exposed to aggregated LDLs, thereby inducing the Wnt/β-catenin pathway [104].

### 4.7. Lectin-like Oxidised LDL Receptor-1 (LOX-1)

Lox-1, a transmembrane protein expressed in endothelial cells, platelets, macrophages, smooth muscle cells and cardiomyocytes, has been implicated in the progression of atherosclerosis, myocardial fibrosis and endothelial dysfunction [105]. Activation of LOX-1 by oxLDL triggers multiple downstream signalling pathways. In a study by Tsai et al. (2016), oxLDL supplementation to HUVECs activated the NF-κB pathway by modulating LOX-1, resulting in increased reactive oxygen species generation that eventually stimulated matrix metalloproteinases (MMPs) [45]. MMPs play a role in vascular tissue remodelling and intimal thickening during vascular calcification in atherosclerosis development [106]. In an in vivo study by Akhmedov et al. (2017), endothelial LOX-1 activation was shown to differentially regulate arterial thrombus formation depending on oxLDL levels. At low oxLDL levels, LOX-1 activates the protective Oct-1/SIRT1 pathway, while it shifts to the thrombogenic ERK1/2 pathway at higher levels [65]. These findings are important, suggesting that the Lox1/SIRT1 pathway may represent a novel therapeutic target for atherosclerosis.

## 5. Future Perspectives

The deleterious effects of oxLDL on various cells have been shown to contribute to inflammation, oxidative stress and impaired vascular homeostasis, all of which accelerate endothelial dysfunction and atherosclerotic plaque formation. As understanding of the signalling pathways underlying atherosclerosis strengthens, the therapeutic potential of non-coding RNAs, including long non-coding RNAs (lncRNAs) and microRNAs (miRNAs), is gaining increasing attention from researchers. These non-coding RNAs have emerged as critical regulators of cardiovascular risk factors and cellular processes, making them promising candidates for enhancing diagnostic and prognostic capabilities in cardiovascular diseases [107].

In a recent study by Yuan et al. (2022), the overexpression of the long non-coding RNA LINC00452 was demonstrated to reverse oxLDL-induced injury in human umbilical vein endothelial cells (HUVECs) by regulating the miR-194-5p/IGF1R axis [108]. Additionally, miR-214-3p has been shown to regulate oxLDL-induced macrophage autophagy, suggesting its potential as a therapeutic target for immune cell function in atherosclerosis [109]. These findings highlight the promise of targeting non-coding RNAs as therapeutic strategies for mitigating oxLDL-induced endothelial damage and immune dysregulation in atherosclerosis. Therefore, future research should focus on further elucidating the interplay between oxLDL, lncRNAs and miRNAs, exploring their roles in endothelial dysfunction and immune responses as well as evaluating their potential as novel biomarkers or therapeutic targets. Therefore, miRNA-based therapies may provide a promising opportunity for clinical intervention, particularly for patients with early-stage or progressing atherosclerosis.

## 6. Conclusions

Oxidised LDL is a key mediator in atherogenesis, driving endothelial dysfunction and contributing to vascular disorders through multiple pro-atherogenic pathways. Its critical role in the initiation and progression of atherosclerosis has been well established, making oxLDL a potential therapeutic target. By gaining a deeper understanding of its atherogenic role and the associated signalling pathways, this study highlights opportunities for the development of novel therapeutic strategies aimed at mitigating the progression of atherosclerosis and improving cardiovascular health outcomes.

## Figures and Tables

**Table 1:** In vitro experimental evidence for oxidised low-density lipoprotein in atherosclerosis.

Cell Type	Experimental Groups and Concentration	Findings	Study
Mononuclear cells			
Human peripheral blood mononuclear cells (hPBMC)	Cells divided into two groups: Group I—normal control Group II—treated with ox-LDL (50 μg/mL)	Compared to normal control group: ↓ Viability ↑ Free radical formation in ROS generation, ↑ inflammation ↑ nuclear translocation of NF-κB p65 ↑ TNF-α, IL-6, VCAM-1 and iNOS mRNA expression	Saji et al. (2018) [38]
Endothelial cells			
Human umbilical vein endothelial cells (HUVECs)	Cells divided into two groups Group 1: untreated control Group 2: treated with oxLDL (100 µg/mL)	Compared to untreated control group: ↑ TNF-α, IL-1β and IL-6 mRNA and protein expression ↑ TLR4 ↑ phosphorylation levels of NF-κB p65 ↑ AKT phosphorylation ↑ ICAM-1 and VCAM-1 ↑ MCP-1 ↑ Adhesion of THP-1	Zhang et al. (2021) [46]
Human umbilical vein endothelial cells (HUVECs)	Cells divided into two groups Group 1: untreated control Group 2: induced with oxLDL (50 µg/mL)	Compared to untreated control group: Activates nuclear translocation of NF-κB p65 subunit ↑ TLR2 and TLR4 mRNA and protein expression ↑ MCP-1 mRNA expression ↑ VCAM-1 & ICAM-1 expression	Bashkar et al. (2016) [47]
Primary mouse lung endothelial cells	Cells divided into two groups: Group 1: untreated control Group 2: treated with oxLDL (100 µg/mL)	Compared to untreated control group: ↑ IKKβ activation ↑ ROS production ↑ NF-κB activation ↑ FAK-dependent ERK activation	Yurgadul et al. (2016) [44]
HUVECs, human umbilical vein endothelial cells (HUVECs)	Cells divided into two groups: Group 1: untreated control Group 2: treated with oxLDL (130 μg/mL)	Compared to untreated control group: NF-κB activation ↑ LOX-1 activation ↑ ROS production ↑ PKC-α activity ↑ MMP-1, MMP-2 and MMP-3 protein ↑ ERK phosphorylation	Tsai et al. (2016) [45]
Human umbilical vein endothelial cells (HUVECs)	Cells divided into two groups: Group 1: untreated control Group 2: induced with oxLDL (100 µg/mL)	Compared to untreated control group: ↑ Cell migration ↑ Apoptosis ↑ Bax protein expression ↑ β-catenin protein expression	Liang et al. (2021) [41]
Human umbilical vein endothelial cells (HUVECs)	Cells divided into two groups: Group 1: untreated control Group 2: treated with oxLDL (100 µg/mL)	Compared to untreated control group: ↓ Viability ↑ Apoptosis ↑mTOR and S6K1a phosphorylation ↑ TNF-α, IL-6, IFN-γ, ↑ inflammation ↓ pAkt expression ↑ ROS production ↓ SOD activity ↓ Nrf2 expression	Zhang et al. (2019) [39]
Primary human umbilical vein endothelial cells (HUVECs)	Cells divided into two groups Group 1: untreated control Group 2: treated with oxLDL (80 μg/mL)	Compared to untreated control group: ↑ Adhesion of THP-1 ↑ VCAM-1, ICAM-1, MCP-1, IL-8 protein expression ↓ p-ERK5, KLF, eNOS	Deng et al. (2018) [34]
Human aortic endothelial cells (HAECs)	Cells divided into two groups: Group 1: untreated control Group 2: treated with oxLDL (50 µg/mL)	Compared to untreated control group: ↑ cell proliferation ↑ Akt phosphorylation	Zhang et al. (2017) [40]
Macrophages			
Mouse RAW264.7 macrophages	Cells divided into two groups: Group 1: untreated control Group2: treated with oxLDL (25 μg/mL)	Compared to untreated control group: ↓ Cell viability ↑ Apoptosis ↑ Cleaved caspase-3 expression ↑ ER stress signalling: ○ ↑ p-PERK, p-IRE1α, p-eIF2α protein expression ○ ↑ ATF4, HERPUD1 mRNA expression	Guo et al. (2018) [49]
Mouse RAW264.7 macrophages	Cells divided into two groups: Group 1: untreated control Group 2: induced with oxLDL (100 µg/mL)	Compared to untreated control group: ↑ NLRP3 inflammasome ↑ IL-1β ↑ LDH level ↑ BRCC36, cleaved caspase-1, NLRP3 proteins ↑ ROS generation	Singh et al. (2019) [50]
Mouse RAW264.7 macrophages	Cells divided into two groups: Group 1: untreated control Group 2: treated with oxLDL (50 mg/L)	Compared to untreated control group: ↑ Nox2, Rac1, p47phox and p22phox expression ↑ Intracellular MDA and ROS production ↓ SOD activity ↑ p38, JNK and ERK1/2 phosphorylation levels	Yang et al. (2015) [51]
Human aorta vascular smooth muscle cells (VSMCs)	Cells divided into two groups: Group 1: untreated control Group 2: treated with oxLDL (100 μg/mL)	Compared to untreated control group: ↑ BSP protein expression ↑ ↑ Runx2	Farrokhi et al. (2015) [52]

Abbreviations: ERK: extracellular signal-regulated kinase; p PERK (the active form of PERK): p-IRE1α (the active form of IRE1α), p-eIF2α and mRNA expressions of ATF4 and homocysteine-inducible; ER stress: inducible, ubiquitin-like domain member 1 (HERPUD1); Runx2: runt-related transcription factor 2; BSP: bone sialoprotein expression.↑: increase ; ↓: decrease.

**Table 3:** Functions and key roles of the signalling pathways affected by oxLDL.

Affected Signalling Pathways	Functions	Key Role	References
Nuclear factor kappa-light-chain-enhancer of activated B-cells (NF-κB)	Regulates cellular stress and mediates immunity by initiating responses to pro-inflammatory signals.	Plays a central role in inflammation by inducing transcription of pro-inflammatory genes.	[66]
Toll-like receptors (TLRs)	Initiates inflammation upon pathogen detection, activating NF-κB and MAPK to produce pro-inflammatory cytokines.	Play a key role in the innate immune system by recognising pathogen-associated molecular patterns (PAMPs) on pathogens like bacteria and viruses.	[67]
Mitogen-activated protein kinase (MAPK)	Relays extracellular signals to regulate diverse cellular programs.	Play an important role in complex cellular programs like proliferation, differentiation, development, transformation and apoptosis.	[68]
Nuclear factor erythroid 2-related factor 2 (NRF2)	Regulates the expression of antioxidant proteins and enzymes.	Protects cardiovascular health by maintaining redox balance and preventing cellular oxidative damage.	[69]
Nucleotide-Binding Domain, Leucine-Rich–Containing Family, Pyrin Domain–Containing-3 (NLRP-3) Inflammasomes	Recognises cellular stress signals and forms inflammasomes.	Crucial for inflammasome activation, leading to cleavage of pro-inflammatory cytokines and amplifying the inflammatory response.	[70]
Wingless/Beta Catenin (Wnt/β Catenin)	The canonical Wnt pathway controls cell proliferation, while noncanonical pathways regulate cell polarity and migration.	Plays an important role in self-renewal during embryonic development.	[71]
Lectin-like Oxidised LDL Receptor-1 (LOX-1)	Mediates oxLDL uptake, leading to foam cell formation and atherosclerosis. Activates oxidative stress and inflammation, causing endothelial dysfunction and cardiovascular disease.	Plays a key role in proatherogenic processes, including macrophage foam cell and plaque formation, endothelial dysfunction, vascular smooth muscle cell proliferation, platelet aggregation and leukocyte recruitment.	[72]

## Data Availability

Not applicable.
